# Prostate cancer patient stratification by molecular signatures in the Veterans Precision Oncology Data Commons

**DOI:** 10.1101/mcs.a006298

**Published:** 2023-12

**Authors:** Kyle M. Hernandez, Aarti Venkat, Danne C. Elbers, John R. Bihn, Mary T. Brophy, Nhan V. Do, Jennifer La, Qiong Liu, Andrew Prokhorenkov, Noah Metoki-Shlubsky, Feng-Chi Sung, Channing J. Paller, Nathanael R. Fillmore, Robert L. Grossman

**Affiliations:** 1 Belay Diagnostics, Chicago, Illinois 60607, USA;; 2 Department of Medicine, University of Chicago, Chicago, Illinois 60637, USA;; 3 Center for Translational Data Science, University of Chicago, Chicago, Illinois 60637, USA;; 4 VA Cooperative Studies Program, VA Boston Healthcare System, Boston, Massachusetts 02130, USA;; 5 Department of Medicine, Harvard Medical School, Boston, Massachusetts 02115, USA;; 6 Chobanian and Avedisian School of Medicine, Boston University, Boston, Massachusetts 02118, USA;; 7 Frederick National laboratory for Cancer Research, Frederick, Maryland 21701, USA;; 8 The Sidney Kimmel Comprehensive Cancer Center, Johns Hopkins University School of Medicine, Baltimore, Maryland 21287, USA;; 9 Department of Medical Oncology, Dana-Farber Cancer Institute, Boston, Massachusetts 02215, USA;; 10 Open Commons Consortium, Chicago, Illinois 60611, USA

**Keywords:** neoplasia of the male external genitalia

## Abstract

Veterans are at an increased risk for prostate cancer, a disease with extraordinary clinical and molecular heterogeneity, compared with the general population. However, little is known about the underlying molecular heterogeneity within the veteran population and its impact on patient management and treatment. Using clinical and targeted tumor sequencing data from the National Veterans Affairs health system, we conducted a retrospective cohort study on 45 patients with advanced prostate cancer in the Veterans Precision Oncology Data Commons (VPODC), most of whom were metastatic castration-resistant. We characterized the mutational burden in this cohort and conducted unsupervised clustering analysis to stratify patients by molecular alterations. Veterans with prostate cancer exhibited a mutational landscape broadly similar to prior studies, including *KMT2A* and *NOTCH1* mutations associated with neuroendocrine prostate cancer phenotype, previously reported to be enriched in veterans. We also identified several potential novel mutations in *PTEN*, *MSH6*, *VHL*, *SMO*, and *ABL1*. Hierarchical clustering analysis revealed two subgroups containing therapeutically targetable molecular features with novel mutational signatures distinct from those reported in the Catalogue of Somatic Mutations in Cancer database. The clustering approach presented in this study can potentially be used to clinically stratify patients based on their distinct mutational profiles and identify actionable somatic mutations for precision oncology.

## INTRODUCTION

Prostate cancer remains the second most common cause of cancer deaths among men in the United States (Siegel et al. 2019). For most men with prostate cancer, definitive local therapy is curative, but for 20%–40% of patients the disease relapses (Paller et al. 2013) and progresses to metastatic castration-resistant prostate cancer (mCRPC) with a median overall survival of 2–3 yr (Armstrong et al. 2020; Tagawa et al. 2022). Since 2010, the therapeutic options for mCRPC have increased quickly, extending survival. New treatments include androgen receptor (AR) signaling inhibitors (enzalutamide, abiraterone, and darolutamide), taxanes (cabazitaxel), radioisotopes (Radium223 and PSMA Lu177), and immunotherapy (sipuleucel-T and pembrolizumab) (Teo et al. 2019). Several genetically targeted therapies have also been approved, including pembrolizumab for prostate cancer patients with microsatellite instability-high (MSI-H) or mismatch repair-deficient (dMMR) cancer (Sokolova and Cheng 2020), and poly(adenosine diphosphate-ribose) polymerase (PARP) inhibitors olaparib and rucaparib for patients with mutations in DNA repair genes, especially *BRCA2* (Marshall et al. 2019). There are many other drugs in development to target new pathways that provide a potential for a more precision-medicine–oriented approach (Frantzi et al. 2020; Yamada and Beltran 2021; Sayegh et al. 2022).

Metastatic prostate cancer has extraordinary heterogeneity in terms of molecular alterations, and this remains a substantial barrier to optimizing patient care and precision oncology for this population (Haffner et al. 2021). Both molecular diversity and heterogeneity point to a major unmet need to understand the molecular biomarkers that can better inform treatment decisions, as well as guide future drug development. A previous study has explored the complex genomic landscape of mCRPC using targeted genomic panels and shown that such patients harbor clinically actionable somatic and germline molecular alterations in PIK3CA/B, RSPO, RAF, APC, β-catenin, and ZBTB16, as well as in genes underlying DNA repair pathways (van Dessel et al. 2019). Additionally, certain mutations, such as *BRCA1*, *BRCA2*, *PALB2*, *CHEK2*, and *ATM*, are more enriched in mCRPC than in primary localized prostate cancer samples, reflecting the unique biology of cancer progression and castration resistance (Robinson et al. 2015; Henríquez et al. 2021).

Veteran Affairs (VA) patients are at increased risk for developing prostate cancer compared with the general population, with a prostate cancer incidence rate ratio of two compared with the nonmilitary population (Zhu et al. 2009). The reason for this increased incidence is unclear, but factors include increased screening rates, exposure scenarios unique to the military such as depleted uranium and Agent Orange, and high rates of STIs (Dennis et al. 2009). In this retrospective study, we use existing clinical data from the VA healthcare system available in Veterans Precision Oncology Data Commons (VPODC) combining clinical data with targeted sequencing (Elbers et al. 2020).

A previous study investigated the genomic landscape of metastatic solid tumors from a sample of the Veterans Health Administration population as part of the National Precision Oncology program, but their focus was not specific to prostate cancers and the sample size for advanced castration resistance was limited (Poonnen et al. 2019a). We show that the VA cohort displays clinically actionable mutations previously reported and potential novel targets. Moreover, we show that unsupervised clustering revealed two subpopulations with novel mutational signatures distinct from existing signatures in the Catalogue of Somatic Mutations in Cancer (COSMIC) database (Tate et al. 2018).

## RESULTS

Somatic targeted sequencing and clinical data from advanced prostate cancer patients available in the VPODC were analyzed using three capture kits. We investigated mutations in genes that were targeted by all capture kits (*N* = 45) and excluded samples with evidence of contamination and poor coverage (*N* = 1), resulting in a total of *N* = 44 samples (Supplemental Table 1; Supplemental Figs. 1, 2). We only considered genes that were targeted by all three capture kits to exclude kit-specific clustering artifacts (Supplemental Fig. 3). As described in more detail below, we classified the veterans cohort into two subgroups using hierarchical clustering and identified two de novo single base substitution (SBS) mutational signatures that did not exhibit strong similarity to existing COSMIC SBS signatures (Methods; Supplemental Fig. 4; Alexandrov et al. 2020).

### Clinical Presentation of the VA Cohort

Table 1 summarizes clinical details, including age at biopsy, biopsy site of sequenced tissue, Eastern Cooperative Oncology Group performance scale, and death status (Supplemental Appendix). Samples were selected as part of the VISN 1 precision oncology program and the VA New England health care system and prioritized for inclusion of patients in the New England region that had advanced disease (Methods; Fiore et al. 2016; Elbers et al. 2020).

**Table 1.** Clinical overview of VA cohort

Characteristics		VPODC Advanced Prostate Cancer Cohort
Count		45
Demographics	Age at results	68.4 (65.6–74.1)
	Race	
	White	36
	Black or African–American	6
	Declined to answer	2
	Unknown	1
	Ethnicity	
	Hispanic or Latino	1
	Not Hispanic or Latino	40
	Declined to answer	2
	Unknown	2
Metastatic status	Yes	41
	No	3
	Unknown	1
Castrate-resistant	Yes	29
	No	4
	Unknown	12
ECOG	0–1	29
	2–4	12
	Unknown	4
Biopsy site	Prostate	31
	Lymph node	5
	Bone	1
	Lung	1
	Soft tissue	1
	Bladder	1
	Gastrointestinal tract	1
	Liver	2
	Brain	1
	Unknown	1
Tumor purity (%)	Min	25.0
	Mean	62.4
	Median	70.0
	Max	90.0
	Unknown (*n*)	7
Deceased status	Yes	43
	No	2

Clinical and demographic characteristics are listed, together with ECOG (Eastern Cooperative Oncology Group) scale. (VPODC) Veterans Precision Oncology Data Commons.

Most patients were identified as metastatic stage (*N* = 41, 91%), and *N* = 29 (64%) were castrate-resistant (Table 1). The metastatic biopsies taken from the prostate were confirmed by the imaging results either recorded directly or summarized in clinical notes provided by the oncologist. *N* = 14 (31%) samples were biopsied from sites other than the prostate. The median pathology estimated tumor purity of this cohort was 70% (range, 25%–90%) across all samples. A small subset of samples were of low tumor purity (<35%, *N* = 5) (Supplemental Table 2). The targeted sequencing libraries were sequenced to a mean target coverage of 717.15× (Supplemental Table 3).

### Somatic Mutations Landscape and *TMPRSS2:ERG* Gene Fusion in Prostate Cancer

**Figure 1.**
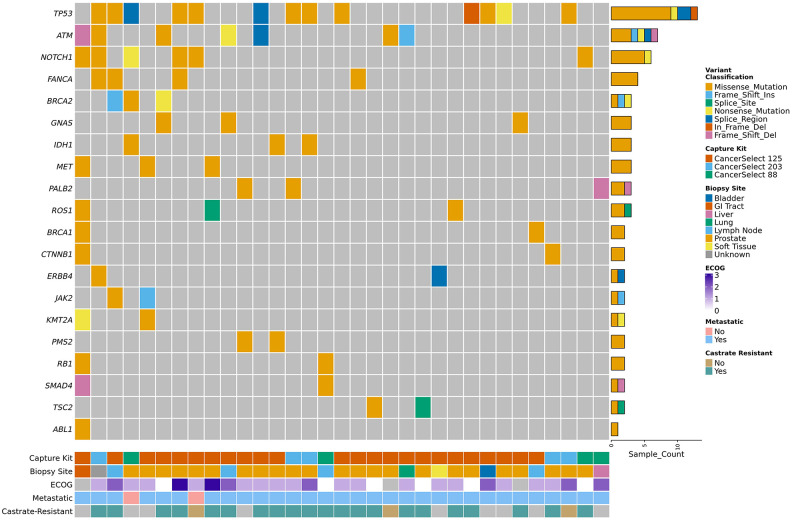
Top 20 mutated genes in the VA Advanced Prostate Cancer Cohort (*N* = 33). *N* = 11 samples lacked mutations in these genes and were excluded from the plot. Patients were ordered based on the frequency of the observed top 20 mutated genes. (ECOG) Eastern Cooperative Oncology Group.

Consistent with previous analysis on metastatic solid tumors in veterans, we found somatic mutations in classic genes such as *TP53*, *BRCA2*, *ATM*, *PALB2*, and *NOTCH1*, with TP53 mutations being the most frequent (Fig. 1; Robinson et al. 2015; Poonnen et al. 2019b; Henríquez et al. 2021). The *TMPRSS2:ERG* gene fusion, which is recognized as one of the most common gene fusions found in prostate cancer patients, was identified in 15 of 44 patients (34%) (Wang et al. 2017), slightly lower than a previously reported estimate of 42.6% in metastatic castrate resistant-prostate cancers (van Dessel et al. 2019). We confirmed the presence of the gene fusion through a visualization of the drastic decline in depth of coverage around the breakpoint (Supplemental Fig. 4).

### Mutation-Driven Clustering and Survival Analyses

First, we used the variant call format (VCF) files to characterize unique mutational signatures in the cohort based on previously reported COSMIC mutational signatures (Manders et al. 2022). The distribution of known COSMIC signatures varied within a sample, and no single signature dominated across samples (Supplemental Fig. 5). Two de novo SBS signatures, defined as mutational signature 1 and mutational signature 2, were identified, reflecting a few unique mutational processes in the cohort (Methods; Supplemental Fig. 6). A heatmap of pairwise cosine similarities between known COSMIC mutational signatures and mutational signatures 1 and 2 shows a moderate level of similarity with COSMIC signature SBS5, but no existing signature exceeded the de novo threshold of 0.85 (Methods; Supplemental Fig. 7). SBS5 has been previously found to be ubiquitous among various cancer types, including prostate cancer (Petljak et al. 2019; Alexandrov et al. 2020).

**Figure 2.**
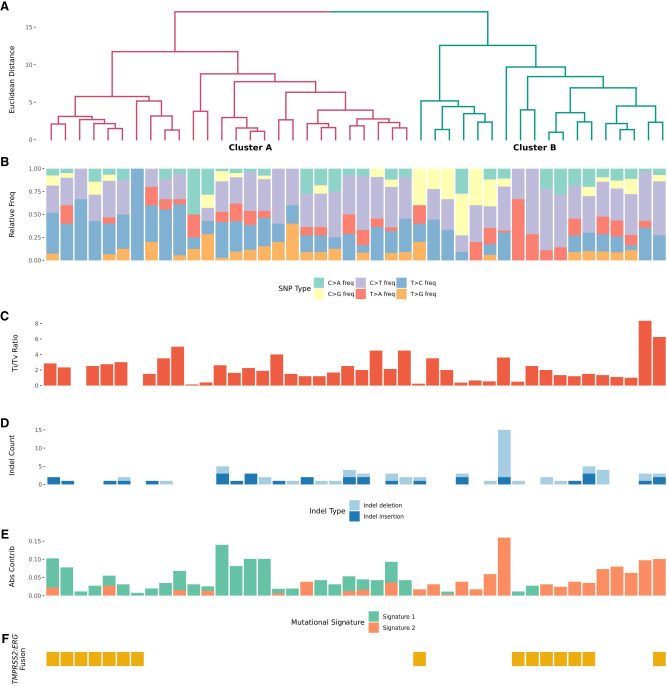
(*A*) Unsupervised clustering revealed two main clusters: Cluster A and Cluster B. (*B*) Relative frequency of SBS changes. (*C*) Transition (Ti) to transversion (Tv) ratio. (*D*) Indel counts. (*E*) Absolute contribution of de novo mutational signatures 1 and 2. (*F*) The presence/absence of *TMPRSS2:ERG* gene fusion.

Next, we performed unsupervised hierarchical clustering on the genomic features extracted from targeted sequencing data to classify the cohort into subgroups (Methods). Two distinct subgroups were identified: Cluster A (*N* = 26) and Cluster B (*N* = 18) (Fig. 2A). A principal component analysis (PCA) showed the separation between these two subgroups using the first three components, with PC1 and PC2 explaining 20.98% and 15.99% of the total variance, respectively (Supplemental Fig. 8).

We inspected the relative frequencies of SBS and counts of indel events and calculated a ratio of transitions (Ti) to transversions (Tv) from SBS frequencies (Fig. 2B–D). Cluster A had a higher Ti/Tv ratio than Cluster B (*x*^2^ = 4.32, df = 1, *P* = 0.04), suggesting the possibility of either difference in the DNA damage mechanism or in DNA repair pathways between the two groups (Fig. 2C). In Cluster B, we noticed an enrichment of mutational signature 2 compared with Cluster A (*x*^2^ = 19.1, df = 1, *P* = 1.3 × 10^−5^) (Fig. 2E). In addition to the patterns of unique somatic mutations of individuals in the two clusters, any differences in sites of biopsies between could also give rise to these differences. Specifically, there were 10 cases with biopsies from outside the prostate that belong to Cluster A, whereas only three belong to Cluster B (Supplemental Table 4). The contribution of nonmetastatic tumors to the clustering pattern appears minimal, with one nonmetastatic prostate biopsy found in Cluster A and Cluster B, each. The TMPRSS2–ERG fusion, a high-frequency fusion gene in prostate cancers, was identified in 34% of the patients (Fig. 2F).

**Figure 3.**
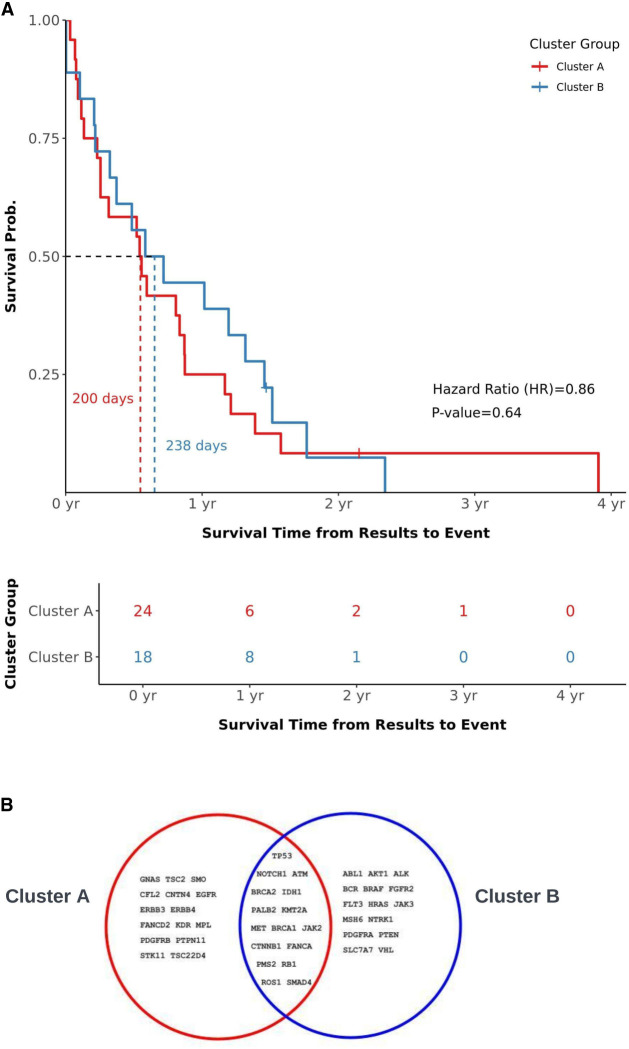
(*A*) The time from sequencing results to death (or censoring) was compared using Kaplan–Meier curves. Number at risk table shows patients who have not yet experienced the death event or were censored at a certain time point. (*B*) Venn diagram shows shared and unique genes in Clusters A and B.

To explore whether there are any differences in survival based on the two clusters, we conducted a Kaplan–Meier (KM) survival analysis. Because any actionable changes in care could be taken after obtaining the sequencing results, we considered the time interval between obtaining sequencing results until death. This analysis suggested a slight difference in survival time, but the results were insignificant (Cluster A 200 d vs. Cluster B 238 d, *P*-value = 0.64) (Fig. 3A). An alternate clinical event that is relevant to advanced prostate cancers is the date of diagnosis of metastatic prostate cancer, but these dates are not available in VPODC. Instead, the date of initial diagnosis of prostate cancer is available, and reanalysis of survival data using these dates yielded insignificant results (data not shown).

### Mutational Analyses of Clusters

Although the low sample size of mutations and our cohort precludes a significant comparison of mutations between Clusters A and B, we examined which mutations were shared or unique to a cluster in a purely descriptive analysis. We found that although most nonsynonymous mutations were common between Cluster A and Cluster B and reflected well-known oncogenes such as TP53, MET, PALB2, and BRCA2, some mutations were unique to one cluster (Fig. 3B). Cluster B included PTEN, a common mutation in mCRPC (Myint et al. 2021), and MSH6, a mismatch repair gene seen in 1% of prostate cancer cases (Bratslavsky et al. 2021). It also included VHL, which is a critical part of the androgen ubiquitination pathway and therefore necessary for many drugs that target increased AR receptor ubiquitination pathway (Kregel et al. 2020). Other genes in Cluster B that can be targeted by agonists and inhibitors include FGFR2, JAK3, and ALK (Table 2; Fig. 3B).

**Table 2.** Genes with approved drug targets

Gene	Action	Drug(s)
*SMO*	Antagonist/inhibitors	Vismogedib Pategedib Taladegib
*GNAS*	Unknown	Dobutamine
*ABL1*	Inhibitors	Bostunib Nilotinib Ponatinib
*MET*	Inhibitors	Tepotinib
*ALK*	Inhibitors	Brigatinib
*FGFR2*	Agonist	Palifermin
*JAK3*	Inhibitors	Decernotinib

Cluster A, on the other hand, included a mutation in *GNAS*, a mutation that has previously been reported as unique to mCRPC and mutually exclusive with AR mutations (Robinson et al. 2015). *GNAS* has no clinically approved drugs or clinical trials; however, it does have several drugs that can potentially act as antagonists (Table 2). Additionally, Cluster A included a mutation in *SMO*, a critical gene in the hedgehog signaling pathway with several potential inhibitors. Hedgehog signaling in the tumor microenvironment can be induced by androgen deprivation and can drive steroidogenesis from benign stromal cells within a prostate tumor (Lubik et al. 2017). An SMO antagonist was able to suppress the development of mCRPC in a xenograft model (Li et al. 2018).

## DISCUSSION

Clinically, prostate cancer is an extraordinarily heterogenous disease. Some patients have slow-growing local disease that only requires observation, whereas others have highly lethal disease even after radical treatments such as surgery, radiation, and castration. This heterogeneity is also apparent at the molecular level, with prostate cancer showing enormous diversity in terms of genetic architecture and intratumoral diversity (Wei et al. 2017). Although it has been demonstrated that genetic signatures predict clinical outcomes more accurately than traditional factors such as tumor stage, PSA level, and Gleason score (The Cancer Genome Atlas Research Network 2015; Gundem et al. 2015), it remains a challenge to optimally stratify prostate cancer patients based on molecular features.

The first-line therapy for metastatic prostate cancer is androgen deprivation therapy and often results in tumor regression. However, it also has the potential to induce castration resistance through mechanisms ranging from AR amplification to increasing AR sensitivity from mutations, as well as activation of pathways that bypass the AR pathway (Gundem et al. 2015). Understanding the molecular evolution and clinical trajectories of prostate cancer remains a challenge and an opportunity, as does connecting this information to clinical care.

Here, we conduct a descriptive analysis of prostate cancer mutations in a small cohort of veterans for whom clinical and genomic data were available in VPODC. We found distinct mutational signatures with clinically actionable targets such as those for *SMO*, *ABL1*, *MET*, *ALK*, *FGFR2*, and *JAK3* (Table 2). Additionally, we identified mutations in *SMAD4/TGF*β (NCT02452008), *ROS1*, *PTEN*, *EGFR*, and *BRAF*, where therapies are currently being explored in the MATCH Trial (NCT02465060). We also identified mutations in *GNAS* that warrant further clinical exploration if confirmed in another larger cohort. Although there was no statistically significant difference in survival between the subpopulations, the small numbers of patients limit any conclusions about the clinical differences in outcomes. Additionally, sequencing tumor-normal matched pairs will allow for a reliable analysis of MSI, homologous recombination deficiency, tumor mutational burden, and copy-number variants—features that were not evaluated in this study. The clustering approach used shows that the different mutational profiles have potential to clinically stratify patients and guide treatment options.

Although not necessarily representative of the entire VA population, our analysis of 45 patients yielded insights consistent with the previous study on prostate cancers in the VA population. We found coding mutations in genes associated with neuroendocrine prostate cancers that were shared by both Clusters A and B, such as *KMT2A* and *NOTCH1*, but not in *PIK3CA* or *KRAS* (Fig. 3B; Poonnen et al. 2019b). This may be due to the unique biology and diversity of advanced metastatic prostate cancers and castration status in veterans, but more follow-up work is needed to test this hypothesis given the small cohort size and rarity of mutations in our data set. The VA cohort has several advantages because it represents a population with diverse ancestry that is offered routine screening and equal access to care. A significant challenge of previous studies has been accessing populations with sufficient diversity to tease out the different contributions of varied ancestry and access to care (Gong et al. 2022). It remains to be seen whether demographic or environmental exposures account for the increased risk of prostate cancer among a military population above and beyond what would be expected from increased screening. Thus, it is possible that tumors in military veterans may have a slightly different evolutionary history compared with a broader population, giving rise to their unique mutational profile.

## METHODS

### Data Sources and Population

This retrospective cohort study was conducted using data from the Veterans Affairs Precision Oncology Data Repository (VA-PODR) available in the VPODC (Elbers et al. 2020). The VA-PODR includes information from four sources: electronic health record (EHR) data from the Corporate Data Warehouse (CDW), manually curated data on cancer cases from the VA Cancer Registry System (VACRS), imaging data, and targeted tumor sequencing information. The study's cohort includes 45 men with prostate cancer for whom targeted tumor sequencing data are available in the VPODC. Data in the VPODC are collected as secondary use data, obtained as part of routine clinical care. Some missing values in VPODC are the result of not all data being properly recorded in the EHR or Cancer Data Warehouse and Cancer Registry. Genomic data used was sequenced as part of clinical care and is made available through consent under the Precision Oncology Data Repository (PODR) (Elbers et al. 2020). All tissues sequenced were formalin-fixed paraffin-embedded (FFPE) tissue specimens. Targeted tumor sequencing was provided through PGDx. Tumor sequencing capture kits were updated as needed during routine clinical care, and several versions used included PGDx CancerSELECT 125 (*N* = 34), PGDx CancerSELECT 203 (*N* = 6), and PGDx CancerSELECT 88 (*N* = 5).

### Sequence Processing and Quality Control

Raw sequencing reads were processed following standard somatic mutation workflows (Zhang et al. 2021). Briefly, reads were aligned to GRCh38 reference available from the Genomic Data Commons (GDC; https://api.gdc.cancer.gov/data/254f697d-310d-4d7d-a27b-27fbf767a834) with BWA 0.7.15-r1142-dirty (Li 2013), PCR duplicates were detected with Picard 2.18.11-SNAPSHOT (http://broadinstitute.github.io/picard/), and base recalibration was applied by GATK v4.0.7.0 (Auwera and O'Connor 2020). The processed alignments were further evaluated for somatic mutations by GATK MuTect2 v4.1.2 (Auwera and O'Connor 2020) in tumor-only mode using a panel of normals (GDC UUID: 6c4c4a48-3589-4fc0-b1fd-ce56e88c06e4) (Zhang et al. 2021). Variants remaining after applying filters from MuTect2 were annotated by variant effect predictor (VEP) v84 (McLaren et al. 2016) using the GDC VEP cache (https://api.gdc.cancer.gov/data/8b9278b3-1e0c-430a-aae5-a944428401c0). Picard v2.22.4 was used to evaluate capture kit-specific coverage to exclude any outliers based on coverage using HsMetrics, and variants located outside of the kit-specific targeted region were removed. Targeted gene lists from each capture kit were intersected to generate a list of common genes. This list was used to include only on-target variants that are present in one of the common genes among capture kits. Cross-sample contamination was assessed with GATK using a 10% threshold. One sample exceeded this threshold and was excluded from subsequent analysis.

### Structural Variant Detection and Annotation

Manta structural variant caller v1.6.0 was used to detect somatic structural variants (SVs) and insertion–deletion mutations (indels) from mapped paired-end sequencing reads (Chen et al. 2016). The predicted SVs were then filtered to reduce false positives using the following criteria: (1) spanning paired-end reads ≥10 or split reads ≥10 and (2) both ends of the inspected SV located within the capture kit intervals. The filtered SVs were then used to detect potential gene fusions using the R package StructuralVariantAnnotation v3.15 (Cameron et al. 2022), with a specific focus on the TMPRSS2:ERG gene fusion. The identified TMPRSS2:ERG gene fusions were then manually investigated with the Arriba v2.3.0 draw fusion.R script (Uhrig et al. 2021).

### Genomic Feature Extraction

The VCF files of all samples across three capture kits were consolidated into a single mutation annotation format (MAF) file using a custom script (https://github.com/uc-cdis/vpodc-prostate-cancer-pub). We then further filtered out the variants with tumor read depth <20. Using VCF files, we computed four genomic features of interest: (1) frequency of six SBSs (C > A, C > G, C > T, T > A, T > C, and T > G); (2) small indel frequencies; (3) de novo mutational signature contributions, defined as those with <85% similarity with previously reported 96 COSMIC SBS; and (4) the presence/absence of the TMPRSS2:ERG gene fusion. The mutational signatures were generated using the R packages MutationalPatterns v3.4.1 (Blokzijl et al. 2018) and NMF v0.24.0 (Gaujoux and Seoighe 2010). Two de novo SBS mutational signatures were detected, and the absolute contribution of each signature was subsequently extracted for each sample.

### Unsupervised Hierarchical Clustering

Unsupervised clustering of samples was performed based on the extracted genomic features using Euclidean distance and Ward's minimum variance method in a hierarchical cluster analysis (Murtagh and Legendre 2014). Cluster assignments were determined by cutting the dendrogram using a Euclidean distance of 15 as a threshold and used for further analysis.

### Matching Genes to Therapy

To investigate therapeutically targetable genes in each cluster, we used the Drug–Gene Interaction Database (https://www.dgidb.org/) (Freshour et al. 2020).

### Survival Analysis

We explored survival from the time when sequencing results were received to the date of death or, for right-censored patients (*N* = 2), the date of the last follow-up. Patients with negative time to the event values (*N* = 2) were removed from the analysis. KM survival analysis was performed using the R package survival v3.4.0 (https://CRAN.R-project.org/package=survival), and survival plots were generated using the survminer R package v0.4.9 (https://CRAN.R-project.org/package=survminer).

## ADDITIONAL INFORMATION

### Data Deposition and Access

Data analyzed in this study are available at https://vpodc.data-commons.org. There are restrictions on the availability of data because of security and privacy considerations. Please refer to the previous VPODC publication for data access guidelines (Elbers et al. 2020).

### Ethics Statement

Additional written consent was not obtained, as all data used in this study were retrieved under Precision Oncology Data Repository agreements and its IRB approval(s), as described in Elbers et al. (2020) (section: “Regulatory Considerations”). For this study, only de-identified data, housed at the Veterans Precision Oncology Data Commons per Data Use Agreement with the Precision Oncology Data Repository, were used.

### Acknowledgments

This work was supported in part by the Open Commons Consortium and the Center for Translational Data Science, University of Chicago. The views expressed are those of the authors and do not necessarily reflect the position or policy of the Department of Veterans Affairs or the U.S. government.

### Author Contributions

Study concept and design were provided by K.M.H., A.V., D.C.E., M.T.B., N.V.D., C.J.P., N.R.F., and R.L.G. Data collection was done by D.C.E., J.R.B., M.T.B., N.V.D., J.L., A.P., F-C.S., and N.R.F. Analysis and interpretation of results were done by K.M.H., A.V., C.J.P., and Q.L. Draft manuscript preparation was done by K.M.H., A.V., D.C.E., J.R.B., Q.L., C.J.P., N.R.F., and R.L.G. System development and operation and management of VPODC were done by K.M.H., N.M-S., and R.L.G.

### Funding

This work was supported in part by the VA Office of Research and Development, Cooperative Studies Program (#CSP2010) and by National Institutes of Health/National Cancer Institute P30 CA006973 (W81XWH-22-2-0024).

### Competing Interest Statement

The authors have declared no competing interest.

### Referees

Majd Al Assaad

Anonymous
